# Microatherosclerosis is a common but often overlooked cause of ischemic stroke

**DOI:** 10.3389/fneur.2026.1920003

**Published:** 2026-10-05

**Authors:** Sucheta Waghmare, Rafael Leal-Hidalgo, Jeffrey L. Saver

**Affiliations:** 1 Department of Neurology, UCLA, Los Angeles, CA, United States; 2 Hospital General Universitario Gregorio Marañón, Madrid, Spain

**Keywords:** ischemic stroke, lacunar stroke, microatherosclerosis, neuropathology, small vessel cerebral disease

## Abstract

**Background:**

Fisher described several distinct vascular pathologies underlying lacunar infarction. However, subsequent literature and clinical practice have often misattributed symptomatic lacunar stroke primarily to lipohyalinosis and intracranial branch ostial atherosclerosis. Classic neuropathologic studies indicate that many lacunar strokes are instead caused by microatherosclerosis - small atheromatous plaque within penetrating arteries. This study aims to quantify the proportion of symptomatic lacunar infarcts in clinical practice attributable to microatherosclerosis.

**Methods:**

Three systematic meta-analyses, per PRISMA guidelines were performed of large clinical series to delineate: (1) the proportion of ischemic strokes due to lacunar infarcts; (2) among lacunar events, the relative frequencies of pure motor hemiparesis (PMH), pure sensory stroke (PSS), dysarthria-clumsy hand (DCH), ataxic hemiparesis (AH); and (3) proportional contribution of microatherosclerosis, lipohyalinosis, intracranial branch-origin atherosclerosis and embolism to symptomatic lacunar stroke.

**Results:**

The neuropathology systematic review showed: PMH caused by microatherosclerosis-64%, lipohyalinosis-14%, embolism-14% and intracranial branch-artery atherosclerosis-7%; PSS caused by lipohyalinosis-100%: and DCH and AH each caused by microatherosclerosis-33%, intracranial branch-artery atherosclerosis-33%, and embolism-33%. The first clinical series meta-analysis showed, among 24 large ischemic stroke series enrolling 32,169 patients, lacunar infarct frequency was 17.8%. The second showed, among 7 large series enrolling 1,646 lacunar infarct patients, subtype frequencies were: PMH-47%, PSS-10.6%, DCH-7.7% and AH-11.2% (with the remainder sensorimotor stroke and less common lacunar syndromes). Quantitative synthesis indicated that: microatherosclerosis is estimated to be the most common cause of lacunar strokes, accounting for 43%, followed by lipohyalinosis-20%, embolism-20%, and intracranial branch-origin atherosclerosis-17%; and that microatherosclerosis is the estimated cause of 7.7% of all ischemic strokes.

**Conclusion:**

Microatherosclerotic plaque within a penetrating artery is a common cause of cerebral ischemia, accounting for an estimated 1 in 13 of all ischemic strokes. This substantial burden of microatherosclerosis may be an important pathophysiologic basis for the benefits of thrombolytic and statin therapy in select patients with lacunar ischemic strokes.

## Introduction

In classic neuropathologic studies, Fisher described four distinct pathophysiologic causes of infarcts in the territory of single, small, deep penetrating arteries: (1) lipohyalinosis; (2) microatherosclerosis; (3) intracranial branch atherosclerosis; and (4) embolism (1, 2). However, subsequent literature and clinical practice have often misattributed symptomatic lacunar stroke primarily to only two of these mechanisms: lipohyalinosis and intracranial branch ostial atherosclerosis (3–13). The need to also recognize embolism as a less common mechanism for lacunar infarcts has been the subject of several studies through subsequent decades, keeping this entity from being completely neglected (14, 15). But microatherosclerosis as a mechanism continues to be often neglected when considering diagnosis and treatment of lacunar stroke. A study estimating the proportion of lacunar strokes due to microatherosclerosis is desirable to firmly restore this concept to neurologic nosology.

The objective of this study was to estimate the frequency of distinct pathophysiologic mechanisms as causes of symptomatic lacunar stroke and all ischemic stroke by performing systematic analyses of: (1) vascular lesions in the classic neuropathologic literature associated with distinct clinical lacunar syndromes; (2) large-scale studies of the frequencies of each distinct clinical lacunar syndrome among all lacunar strokes; and (3) large-scale studies of the frequency of lacunar stroke among all ischemic strokes. A particular focus was upon intrinsic small vessel microatherosclerosis because of its historical eliding in discussions of lacunar stroke pathogenesis.

## Methods

As this study analyzed publicly available, de-identified data from previous studies, it was exempt from UCLA IRB approval and did not require informed consent.

### Meta-analyses general methods

We performed three meta-analyses to determine: (1) the frequency of lacunar stroke among all ischemic strokes; (2) the relative frequencies of classical lacunar stroke clinical syndromes among all lacunar strokes; and (3) the pathologically-identified mechanisms of lacunar stroke associated with each lacunar clinical syndrome, with special attention to microatherosclerosis. Detailed methods for each meta-analysis are provided in the Supplementary eMethods text, with the general approach taken for all three briefly delineated here.

The meta-analyses adhered to the Preferred Reporting Items for Systematic Reviews and Meta-Analyses (PRISMA) guidelines (16). Formal searches were conducted in PubMed to identify studies published through July 2024 (17). The formal search strategies for each meta-analysis are sown in the Supplementary eMethods text. Reference lists of retrieved articles and previous systematic reviews were screened for additional relevant studies. The study protocol was not prospectively registered.

The quality of included observational studies was assessed using the ROBINS-E tool. This assessment evaluated seven key domains for potential bias due to: confounding, participant selection, arising from measurement of exposure, post-exposure interventions, missing data, measurement of outcome, selection of reported results. All studies identified in systematic PubMed searches were screened by one reviewer (SW) and a second reviewer (JLS) further screened studies deemed probably or possibly salient by the first reviewer, with the final decision by consensus if disagreement occurred. Two investigators (SW and JLS) independently extracted data from the included studies. Discrepancies between reviewers were resolved by a re-review and consensus discussion.

The quality of included observational studies was assessed using the ROBINS-E tool (18). Each study was rated as having low, moderate, serious, or critical risk of bias by two independent raters (SW and JLS) with discrepancies resolved through discussion. The overall risk of bias was determined by the highest level of bias identified across all domains.

### Meta-analysis 1: frequency of lacunar strokes among all ischemic strokes

Criteria for were: (1) formal classification of ischemic stroke subtypes in consecutive patients performed using any of 3 validated classification systems: TOAST (Trial of ORG 10172 in Acute Stroke Treatment); ASCOD (atherosclerosis, small vessel disease, cardiac pathology, other causes, and dissection); or Causative Classification System (19–21) and (2) studies reported the frequencies of each of five causes: large artery atherosclerosis, SVD, cardioembolism, other defined cause, and undetermined/cryptogenic.

### Meta-analysis 2: frequency of classical lacunar syndromes among all lacunar strokes

Criteria for inclusion in the study of the relative frequencies of classical lacunar stroke clinical syndromes among all lacunar strokes were: (1) moderate to large (≥ 10 patients) clinical series of consecutive patients; (2) reporting of the proportion of lacunar strokes due to pure motor hemiparesis, pure sensory stroke, dysarthria-clumsy hand, and/or ataxic hemiparesis. Exclusion criteria included studies focusing on non-lacunar stroke subtypes, studies not providing information on the syndromic subtypes of lacunar stroke, or those without original data (e.g., review articles).

### Meta-analysis 3: Pathologically Identified Causes of Lacunar Stroke

An additional meta-analysis was performed to quantify the pathologically-identified mechanisms of lacunar stroke associated with each lacunar clinical syndrome, with particular attention to the role of microatherosclerosis. Any mechanism mentioned once or more in the studies was included as a pathophysiologic category, which resulted in five distinct categories (Figure 1 and Table 1):

(1) Lipohyalinosis: refers to a small-vessel vasculopathy involving penetrating arteries typically 40–200 μm in diameter^2^. It is characterized by fibrinoid necrosis, hyaline and lipid deposition, and mural thickening. Different sources identify lipohyalinosis as identical to, partially overlapping with, or different from arteriolosclerosis. For this study, we considered arteriosclerosis a subset of lipohyalinosis, present when the affected vessel was in the size range of arterioles (10–100 μm).
(2) Microatherosclerosis: refers to atheromatous plaque formation within penetrating arteries typically 200–800 μm in diameter^2^. The stages of atherosclerotic plaque development include: adaptive intimal thickening; fatty streak; atheroma; fibroatheroma or atheroma with thick fibrous cap; and complicated plaques with surface defects and/or hematoma, hemorrhage, and/or thrombosis (22). A defining feature of microatherosclerosis is that the plaque is confined entirely within the penetrating artery and does not extend into the parent artery.
(3) Intracranial branch ostium atherosclerosis: refers to the development of atherosclerotic plaque at the junction where a small penetrating artery arises from a larger parent intracranial vessel (23). This process leads to narrowing or occlusion of the branch ostium, impairing distal perfusion.
(4) Embolism: refers to occlusion of a penetrating artery by material (usually a thrombus) originating from a distant site, such as a proximal artery or the heart and typically involves larger penetrating arteries (200–850 μm)^2^.

**Figure 1:**
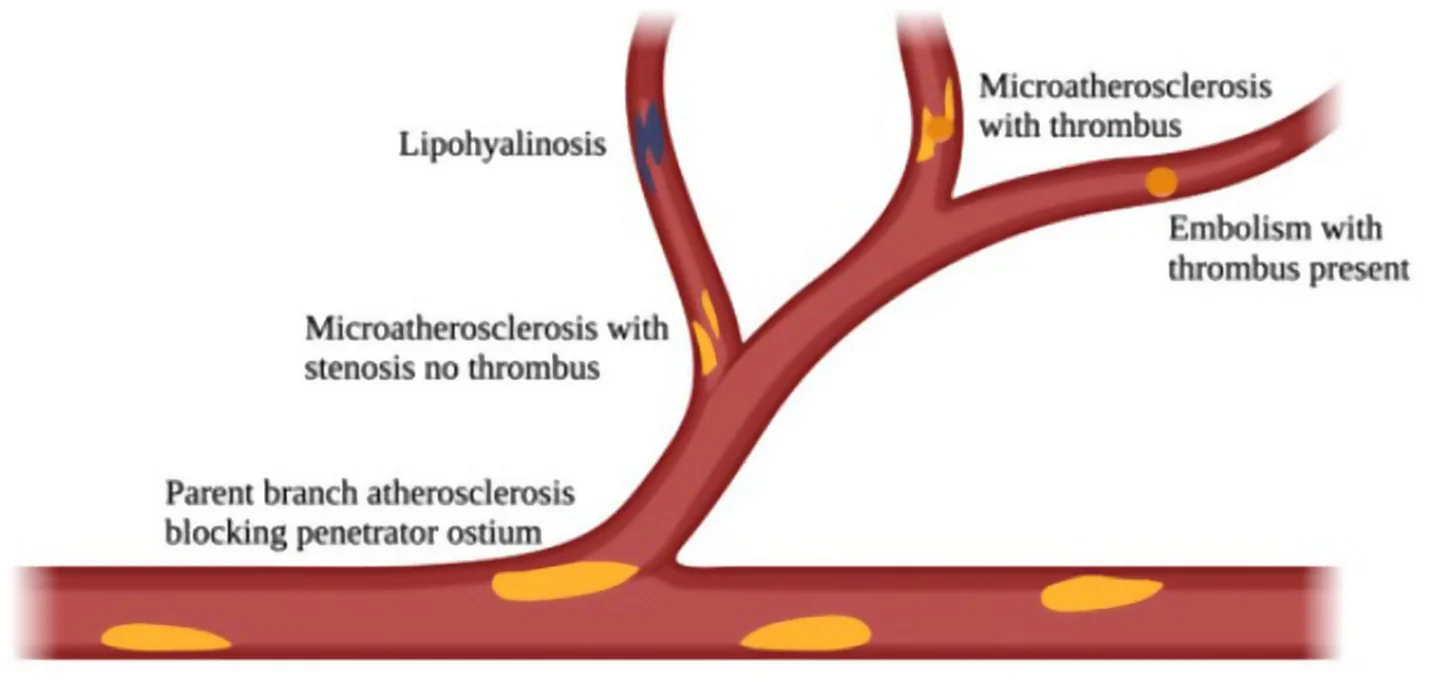
Schematic representation of the four leading cerebral vessel pathologies associated with lacunar infarction. (1) Microatherosclerosis in penetrating arteries with radial diameters 200 to 800 μm; (2) Lipihyalinosis, in smaller penetrating arteries and arterioles with radial diameters of 40–200 μm; (3) Large artery atherosclerosis in parent vessels with radial diameters of 1.25 mm to 7 mm, blocking penetrator ostia; and (4) Embolism, typically occluding larger penetrating arteries, 200–850 μm.

**Table 1:** Pathologic definitions of lacunar stroke substrates.

Lacunar stroke substrates	Vessel size	Pathologic definition
Lipohyalinosis	40–200 μm	A small-vessel vasculopathy characterized by fibrinoid necrosis, hyaline and lipid deposition, and mural thickening. Arteriosclerosis was considered a subset of lipohyalinosis when the affected vessel was in the arteriole size range (10–100 μm).
Microatherosclerosis	200–800 μm	Atheromatous plaque formation within a penetrating artery, confined entirely within the vessel and not extending into the parent artery.
Intracranial branch ostium atherosclerosis	Branch ostium	Atherosclerotic plaque at the junction where a penetrating artery arises from a larger parent intracranial vessel, causing narrowing or occlusion of the branch ostium and impairing distal perfusion.
Embolism	200–850 μm	Occlusion of a penetrating artery by material, usually a thrombus, originating from a distant site such as a proximal artery or the heart.

Eligible studies met the following inclusion criteria: (1) postmortem neuropathologic studies providing detailed analysis of arterial vessel pathology in a series (two or more) of patients with lacunar stroke associated with one or more of the five cardinal lacunar syndromes (pure motor hemiparesis, pure sensory stroke, dysarthria-clumsy hand, ataxic hemiparesis, and sensorimotor stroke). Exclusion criteria included studies lacking vessel analysis, focusing on non-lacunar stroke subtypes, single case reports, or those without original data (e.g., review articles). Study selection was performed in two stages, with abstracts screened initially and full texts reviewed for final inclusion by two independent reviewers. Discrepancies were resolved through discussion.

While neuropathologic studies of the lesions underlying PMH, PSS, DCH, and AH were identified, no study was found for SMS. Imaging studies have indicated that sensorimotor stroke can arise not only from single, penetrating artery infarcts but also infarcts of multiple, adjoining penetrating arteries with ostial occlusion due to parent vessel thromboembolic occlusion (24–31). Accordingly, for the epidemiologic projections, half of SMS cases were considered to be lacunar (single penetrator) in nature, with sensitivity analyses performed for a lower bound of 30% and upper bound of 60%. As the infarcts in single penetrator SMS are larger in size, similar to those of PMH, the SMS single penetrator infarcts were projected to have similar rates of causative lesions as PMH. Also, no study was found for OTH. To minimize assumptions, patients with OTH clinical syndromes were projected have the same proportion of causative vascular lesions as for the 4 classic lacunar syndromes. Sensitivity analyses were performed of three extreme distributions in which OTH clinical syndromes were due to: (1) the same causes of PMH only; (2) the same causes of PSS only; and (3) the same causes of combined DCH/AH only.

### Statistical analyses

To compute the aggregate prevalence estimate in Meta-Analysis 1, we employed a DerSimonian–Laird random-effects model, applied to logit-transformed proportions (with 0.5 continuity correction), and back-transformed to the proportion scale. Heterogeneity was assessed using the I^2^ statistic. First drafts of forest plots were constructed using Claude (Anthropic) with results assessed and corrected as needed by the authors. The aggregate frequency estimates in Meta-Analyses 2 and 3 were derived using study sample-size weighted means, with study-level means performed as a sensitivity analysis. As the analysis assessed proportion values for six syndrome subtypes (Analysis 2) and four neuropathologic subtypes (Analysis 3) concurrently, rather than single prevalence variables, more formal meta-analytic analyses were not performed. Confidence intervals for pooled frequency estimates were calculated by converting each variable’s confidence interval into a percentage margin of error, combining those percentages using the square-root-of-sum-of-squares rule, and then applying that combined percentage to the multiplied point estimate using logit-transformed proportions. Figure 2 schematically shows the overall analytic data flow of the study.

**Figure 2:**
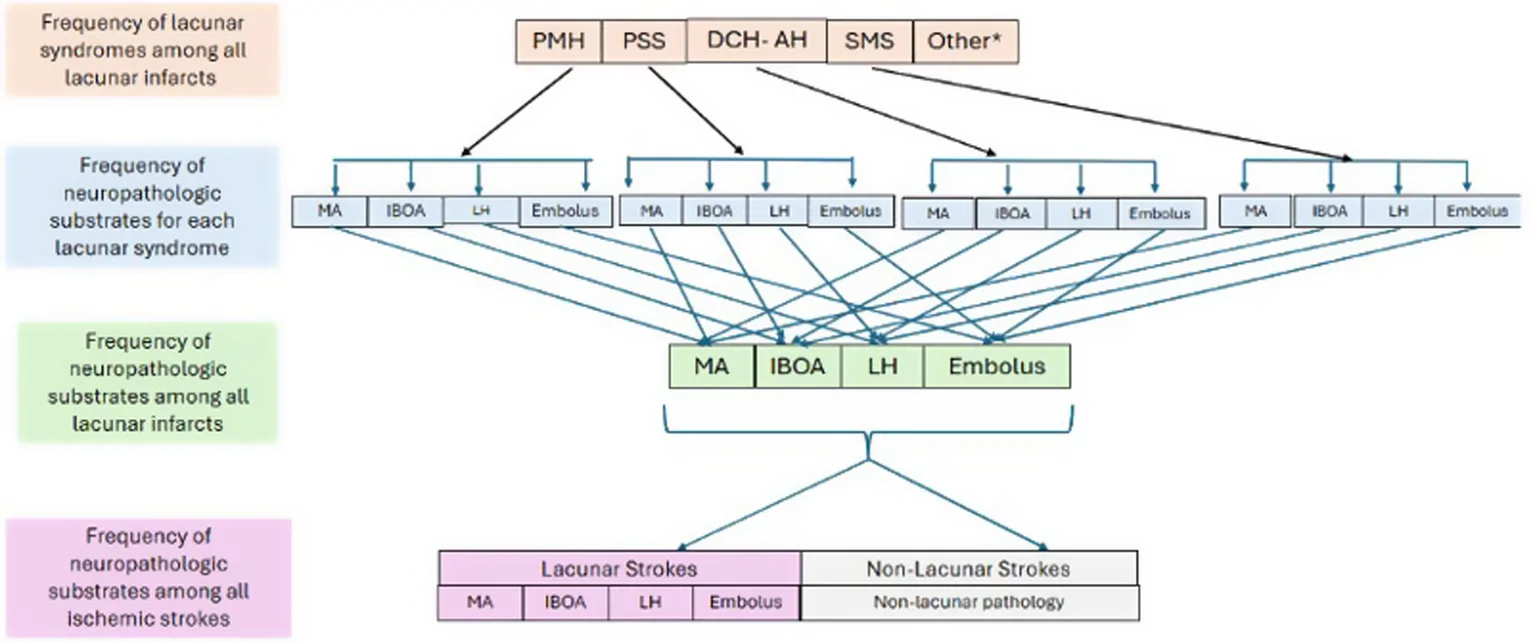
A graphical summary illustrating the integration of the three meta-analyses into the final estimates * The “Other” category was treated similarly to the PMH, PSS, DCH-AH, and SMS groups but is not shown because of space constraints.

Since the systematic review of neuropathologic studies identified only a very limited number of fully analyzed cases, all study findings should be considered exploratory rather than definitive. None of the retrieved studies overlapped in enrolled populations.

## Results

### Frequency of lacunar stroke among all ischemic strokes

For the analysis of the frequency of lacunar strokes among all ischemic strokes, Figure 3A shows the flow of article selection. The systematic search yielded 118 records, among which 94 were excluded as not fulfilling criteria selection criteria. The leading reasons for exclusion were: study reported patients with nonconsecutive ischemic stroke (*n* = 36); and studies without formal ischemic stroke subtype classification (*n* = 15). A total of 24 reports enrolling 7,700 individuals were identified as fully meeting entry criteria.

**Figure 3:**
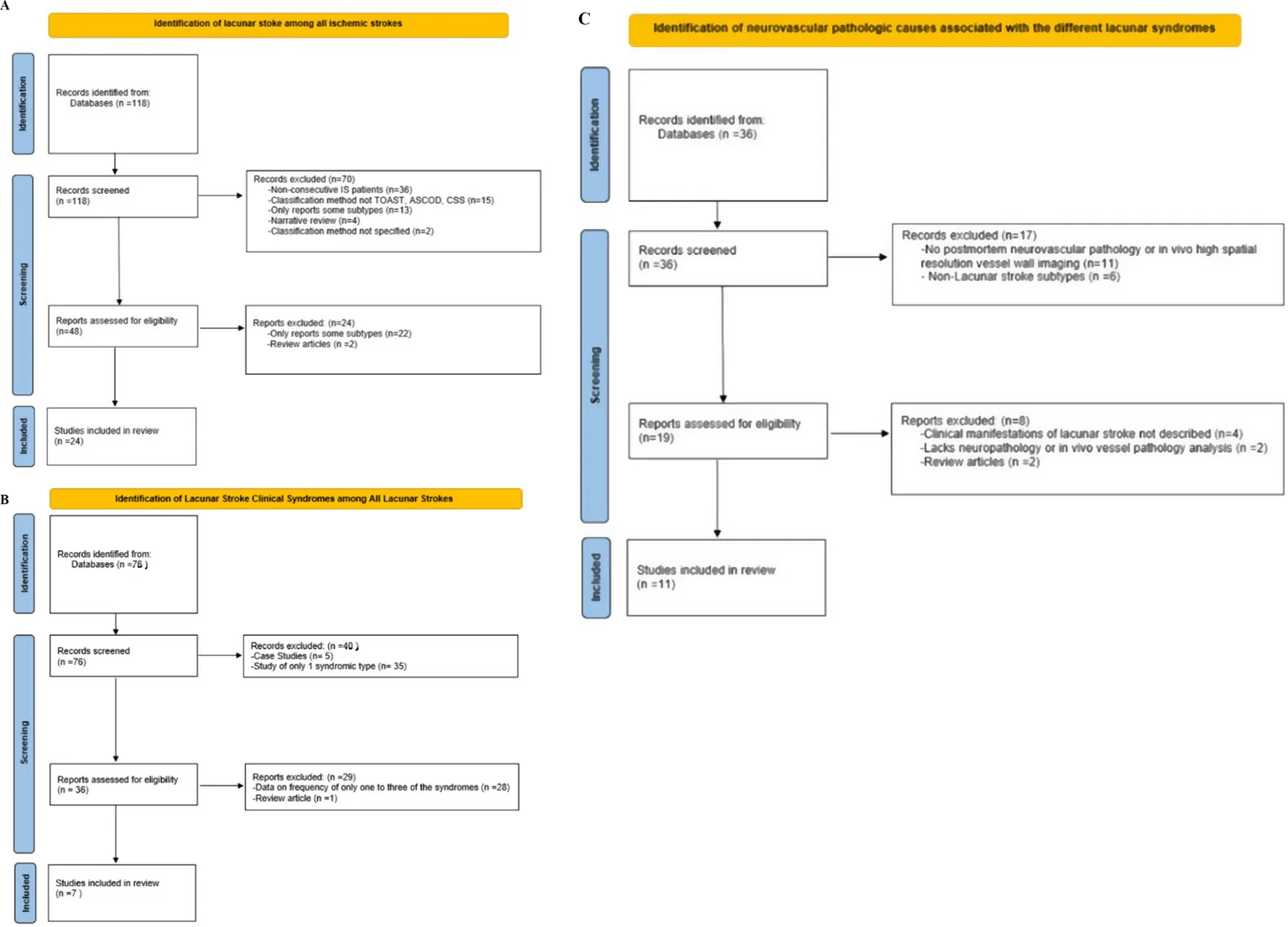
PRISMA flow diagrams showing study selection flow for: **(A)** frequency of lacunar strokes among all ischemic strokes, **(B)** frequency of each of the lacunar clinical syndromes among all lacunar strokes; and **(C)** neurovascular pathologic causes associated with the four classic lacunar syndromes. PRISMA: Preferred Reporting Items of Systematic Reviews and Meta-Analyses.

Initial forest plot meta-analytic aggregation of these reports identified one study as an extreme outlier and it was excluded from the lead analysis (32). Combined analysis of the resulting 23 studies is shown in Figure 4. Heterogeneity was substantial at I^2^ = 92.6% (A forest plot including the outlier study is shown in Supplementary Figure S1. The point estimate increased to 18.75 [95% CI, 14.4–23.9) and the heterogeneity increased to I^2^ = 99.1%.

**Figure 4:**
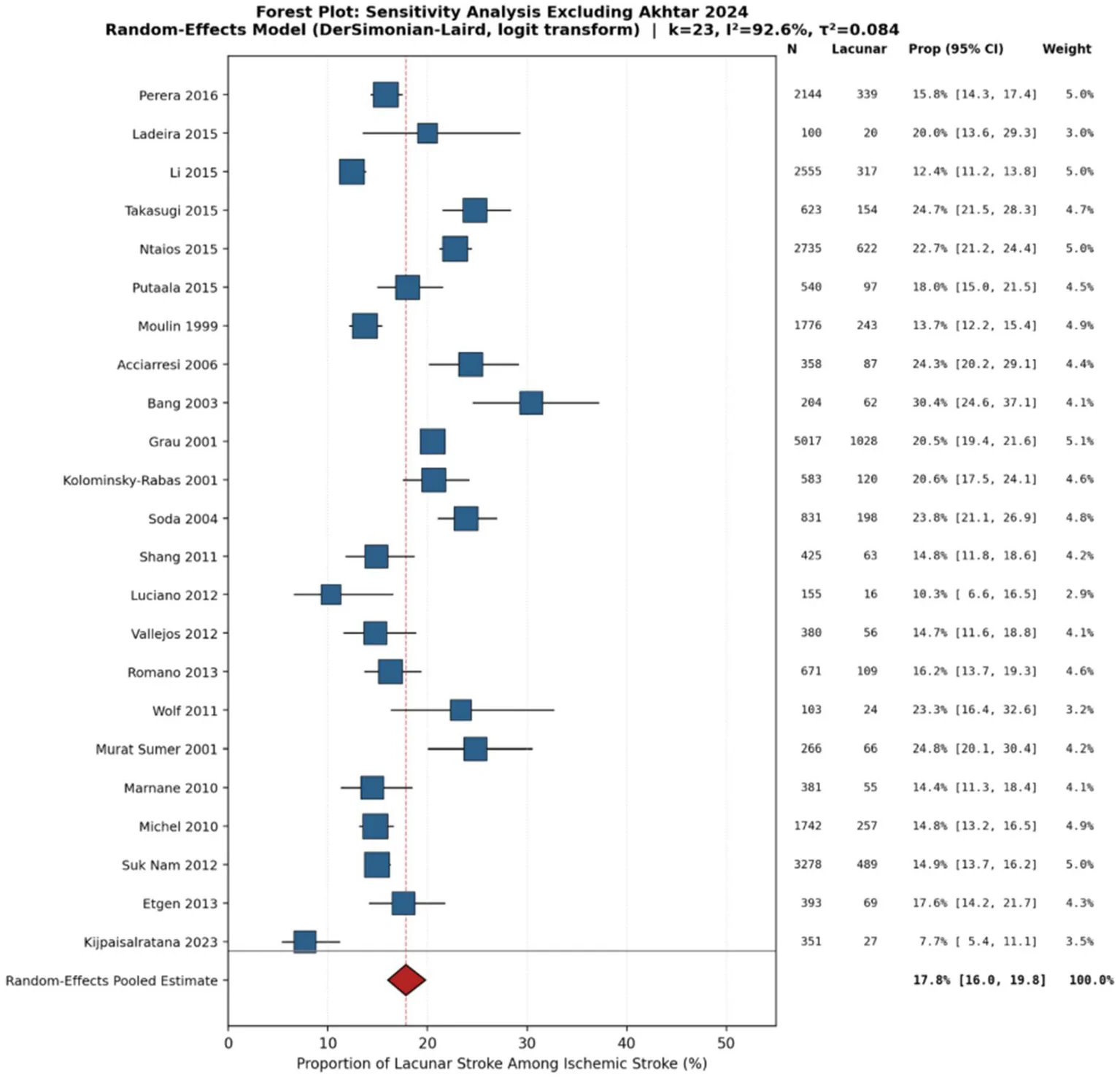
Forest Plot demonstrating Proportion of Lacunar Stroke Among All Ischemic Stroke. Random-Effects Model (DerSimonian–Laird, logit transform) | k = 23, I^2^ = 92.6%. Random-effects pooled estimate 17.8% (95% CI, 16.0–19.8). Akhtar et al. (32) excluded as a statistical outlier.

The risk of bias analysis results is shown in Supplementary Table S1. Among the 24 observational studies, no risks were noted in the categories of Participant Selection, Measurement of Exposure, Post-exposure Intervention, or Selection of Reported Results. Some concerns were present for 6 studies in Confounding; and some/high concerns n 17 studies for Missing Data, generally due to differences in stroke subtype classification or incomplete records. The issues were limited in scope and unlikely to substantially influence overall findings.

### Frequency of lacunar stroke clinical syndromes among all lacunar strokes

For the analysis the frequency of each of the lacunar clinical syndromes among all lacunar strokes, Figure 3B shows the flow of article selection. The systematic search yielded 76 records, among which 40 were excluded based on study titles or screening of study abstracts, and 29 further records were excluded based on detailed screening of full text (Figure 2B). The leading reasons for exclusion were: study reported only a single syndromic subtype (*n* = 35); study reported data on only one to three of the target syndromes (*n* = 28); case series/report (*n* = 5); and review article without original data (*n* = 1) A total of seven studies describing 1,646 individuals were identified as fully meeting study selection criteria.

The findings of the seven studies and the study-level and sample-size weighted means for frequencies of each lacunar stroke clinical syndrome among all lacunar strokes are shown in Table 2. The study-level mean and sample-size weighted frequencies of the lacunar stroke clinical syndromes were similar. The study weighted frequencies were: pure motor hemiparesis (47%), pure sensory stroke (10.6%), dysarthria clumsy hand syndrome (7.7%), ataxic hemiparesis (11.2%), other (8.8%), and sensorimotor stroke due to lacunar infarct (15.7%), as seen in Supplementary Figure S1. The number of dysarthria clumsy hand is projected based on ratio of DCH to AH in other studies.

**Table 2:** Frequency of classical lacunar stroke clinical syndromes among patients with lacunar stroke in individual studies.

Study*	Lacunar strokes (N)	PMH	PSS	DCH	AH	Other	SMS-LI
Arboix et al. (25)	566	256 (45.2%)	105 (18.5%)	50 (8.8%)	20 (3.5%)	66 (11.6%)	69 (12.1%)
Chamorro et al. (35)	316	181 (57.2%)	21 (6.6%)	18 (5.6%)	33 (10.4%)	0 (0.0%)	63 (19.9%)
Besson et al. (33)	200	77 (38.5%)	14 (7%)	17 (8.5%)	38 (19%)	37 (18.5%)	17 (8.5%)
Micheli et al. (34)	196	104 (53%)	9 (4.5%)	0 (0.0%)	34 (17.3%)	0 (0.0%)	49 (25%)
Hommel et al. (29)	100	35 (43.8%)	11 (13.8%)	8 (32.5%)	18 (10.0%)	21 (21.0%)	7 (7.0%)
Gan et al. (36)**	225	101 (44.8%)	15 (6.6%)	15 (6.6%)	41 (18.2%)	7 (3.1%)	46 (20.4%)
Schonewille et al. (37)	43	20 (46.5)	1 (2.3%)	2 (4.6%)	11 (25.5%)	0 (0.0%)	9 (20.9%)
Total N	1,646	774	176	128	185	131	260
Mean frequency—patient-level		47.0%	10.6%	7.7%	11.2%	8.8%	15.7%
Mean frequency—study-level		45.0%	6.8%	9.5%	14.8%	7.7%	16.2%

PMH, pure motor hemiparesis; PSS, pure sensory stroke; DCH, dysarthria-clumsy hand; AH, ataxic hemiparesis; SMS-LI, sensorimotor stroke-lacunar infarct, OTH, other.

*Article citations for studies in Supplement.

**Gan et al included DCH in the Other category. Number of DCH re-binned projected based on ratio of DCH to AH in other studies.

The risk of bias analysis results are shown in Supplementary Table S2. Overall, the studies demonstrate robust methodological quality, with low risk of bias across key domains including participant selection, exposure measurement, post-exposure interventions, missing data, and selective reporting. Confounding was effectively addressed in nearly all studies, with only minimal concerns noted for a single study.

### Frequency of pathologically identified causes of lacunar stroke

For the analysis the frequency of each of the neurovascular pathologic causes associated with the different lacunar syndromes, Figure 3C shows the flow of article selection. The systematic search yielded 36 records, among which 17 were excluded based on study titles or screening of study abstracts, and 8 further records were excluded based on detailed screening of full text (Figure 2C). The leading reasons for exclusion were: absence of postmortem neurovascular pathology or *in vivo* high-spatial-resolution vessel-wall imaging (*n* = 11); and non-lacunar stroke subtypes (*n* = 6) A total of 11 studies describing 20 symptomatic lacunar strokes were identified as fully meeting study selection criteria.

The clinical syndrome with the most cases in which the neurovascular substrate was identified was pure motor hemiparesis, with 14 cases. All of the others had sparse numbers: pure sensory stroke – 2; dysarthria-clumsy hand/ataxic-hemiparesis – 3; sensorimotor stroke – 1. Supplementary Table S3 shows the frequencies with which different neurovascular pathologies were associated with each clinical syndrome. For pure motor hemiparesis, the most common substrate was microatherosclerosis in 64%, with infrequent representation of lipohyalinosis, intracranial branch ostium atherosclerosis, and embolus. All cases of pure sensory stroke were lipohyalinosis. DCH/AH cases were equally divided among microatherosclerosis, intracranial branch ostium atherosclerosis, and embolus. For the sensorimotor stroke case, the neurovascular pathologic findings were inconclusive.

In addition to these findings in 20 patients with symptomatic lacunar infarcts, neurovascular pathologic findings were also reported for 50 predominantly asymptomatic lacunar infarcts (38). The 50 infarcts were studied in four patients, only one of whom clearly had a clinical lacunar syndrome (pure motor hemiparesis) associated with one of the harbored lesions. Among these 50 infarcts, the neurovascular pathologic substrate was lipohyalinosis in 43 (86%), thrombosis of a fusiform microaneurysm in 2 (4%), compressive pressure from a nearby microaneurysm in 1 (2%), microatherosclerosis in 1 (2%), potential embolus in 1 (2%), and inconclusive in 4 (8%).

### Frequency of vascular pathologic causes of symptomatic lacunar and all ischemic stroke

The results of the three systematic reviews were integrated to delineate the frequencies of the different causes of symptomatic lacunar ischemic stroke (Table 3) The most common substrate was microatherosclerosis, estimated at 43.0%. Estimates for each of the three other mechanisms, lipohyalinosis, intracranial branch ostium atherosclerosis, and embolism each accounted for approximately 20% of lacunar strokes. Sensitivity analyses for the assumptions related to SMS and OTH lacunar clinical syndromes did not substantially alter the results (Supplementary Tables e4a, e4b, e5a, e5b). Given that 17.8% of all ischemic strokes were found to be lacunar, among all ischemic strokes, microatherosclerosis was estimated to account for 7.7% of all ischemic strokes, lipohyalinosis for 3.5%, intracranial branch ostium atherosclerosis for 3.0%, and embolism for 3.6%.

**Table 3:** Frequencies of neuropathologic substrates among lacunar strokes and all ischemic strokes.

Lacunar sroke syndrome		Clinical syndrome frequency among all lacunar strokes	Frequencies of neuropathologic substrates underlying Each lacunar infarct syndrome*			
			Microathero-sclerosis	Lipohyalinosis	Intracranial branch ostium atherosclerosis*	Embolism
Lacunar stroke syndrome	PMH	47.0% (44.6%–49.4%)	64%	14%	7%	14%
	PSS	10.6% (9.2%–12.2%)	0.0%	100%	0.0%	0.0%
	DCH	7.7% (6.5%–9.1%)	33%	0.0%	33%	33%
	AH	11.2% (9.7%–12.8%)	33%	0.0%	33%	33%
	Other	8.8% (7.5%–10.2%)	25.0%	25.0%	25.0%	25.0%
	SMS-LI	15.7% (14.0%–17.6%)	33%	0.0%	33%	33%
Frequency of neuropathologic substrate among all lacunar ischemic strokes			43.0% (40.6%–45.4%)	19.7% (17.8%–21.6%)	17.1% (15.3%–18.9%)	20.2% (18.3%–22.1%)
Frequency of neuropathologic substrate among all ischemic strokes**			7.7% (7.4%–8.0%)	3.5% (3.3%–3.7%)	3.0% (2.8%–3.2%)	3.6% (3.4%–3.8%)

PMH, pure motor hemiparesis; PSS, pure sensory stroke; DCH, dysarthria-clumsy hand; AH, ataxic hemiparesis; OTH, other; SMS-LI, sensorimotor stroke-lacunar infarct.

* Table values in parentheses are 95% confidence intervals; Supplementary Table S3 shows the raw number values underlying these frequencies.

**Based upon meta-analytic estimate that lacunar infarcts account for 17.8% of all ischemic strokes.

## Discussion

In this study, systematic meta-analyses of multiple data sources and data synthesis were performed to estimate the frequencies of the different major vascular pathologic causes of symptomatic lacunar stroke. Microatherosclerosis was the most common substrate, estimated to account for more than 4 of every 10 symptomatic lacunar strokes, while lipohyalinosis, intracranial branch ostium atherosclerosis, and embolism were each estimated to account for approximately 2 of every 10. Considered from the larger framework of all ischemic strokes, microatherosclerosis was estimated to be cause of nearly 1 in 12, while lipohyalinosis, intracranial branch ostium atherosclerosis, and embolism were each estimated to account for approximately 1 in 25.

These results challenge an understanding of the pathogenesis of symptomatic lacunar stroke that commonly prevails in the research literature, neurologic textbooks, and clinical practice. Frequently authorities state that lipohyalinosis and intracranial branch atheromatosis are the predominant cause of symptomatic lacunar stroke, with and embolism occurring less frequently, and microatherosclerosis not recognized at all (3–12). This view is incorrect and reflects a misreading of Fisher’s classic neuropathologic studies that has persisted over decades. It is important to emphasize that several investigators and authors have more accurately recognized microatherosclerosis as an important mechanism of symptomatic lacunar stroke (39, 40). But misunderstanding has been widespread.

The genesis of this longstanding misreading likely has several sources: (1) As Fisher was the only senior investigator ever to perform detailed pathologic studies of penetrating arteries, there was not a community of experienced investigators to recognize and correct missteps. (2) Fisher reported his findings in a manner that was richly descriptive and insightful, but more qualitative than quantitative. (3) Lipohyalinosis was the most common cause pathologic lacunar infarcts in Fisher’s studies, but almost all due to LH were small and asymptomatic; the high frequency of LH findings in asymptomatic lesions obscured its much reduced association with symptomatic, lacunar infarcts typically of larger size. (4) Just as Fisher completed his work, new technologies of CT and MR imaging arose, shifting investigative focus away from neuropathologic studies. (5) Caplan and Fisher introduced the term “intracranial branch atheromatosis” to describe lacunar strokes due to occlusion of penetrating artery ostiums by atherosclerosis in larger, parent arteries. But this term lacked specificity and could be interpreted as encompassing both ostial atherosclerosis and intrinsic small vessel microatherosclerosis. We have therefore here revised this rubric to the more specific “intracranial branch origin atheromatosis.” (6) While atherosclerosis is a common mechanism of ischemic injury in many organs and arteriolosclerosis a major mechanism of kidney disease, the rubric lipohyalinosis has only been applied to cerebral arteries. Accordingly, invoking lipohyalinosis as a mechanism provided neurologists with a clinical perspective unavailable to other specialties that enabled them to provide distinctive professional insights and care.

The current study is perforce consistent with the prior literature as it is based on systematic meta-analyses of prior investigations. But it is the first to our knowledge to integrate information from series delineating the frequency of clinical lacunar syndromes with the neuropathologic data specific to symptomatic lacunar stroke, rather than combined asymptomatic and symptomatic lacunar infarcts. Consequently, the present investigations extends Fisher’s original work and that of later investigators who correctly understood his studies (refs) by providing for the first time quantitative estimates of the causative frequencies of each of the major mechanisms of symptomatic lacunar stroke.

There may be important therapeutic implications arising from the diversity of causes of lacunar ischemic stroke. Preventive therapies targeting atherosclerosis, including lipid-lowering agents, would be expected to be beneficial for patients with ischemic strokes due to microatherosclerosis or intracranial branch ostium atherosclerosis. In contrast, it is uncertain pathophysiologically if lipid-lowering agents would enhance prevention in patients with lipohyalinosis, although of course treatment of hypertension would be essential and perhaps should have particularly aggressive BP-lowering targets. With regard to acute treatments, thrombolytic agents would be expected to be beneficial for patients with ischemic strokes due to microatherosclerosis or intracranial branch ostium atherosclerosis, among whom supervening *in situ* thrombosis is a frequent mechanism. Thrombolytics would of course also be beneficial for emboli that have lodged within small vessels. Whether *in situ* thrombosis is a mechanism of stroke in patients with lipohyalinosis is less certain from neuropathologic studies. However, patients with severe lipohyalinosis likely will sometimes develop very slow flow in distal artery segments leading to stasis clotting, which would also benefit from thrombolytic treatment.

This study has limitations. First, though large numbers of studies and patients contributed to the meta-analysis of the relative frequencies of different lacunar stroke clinical syndromes and the frequency of lacunar stroke among all ischemic strokes, the number of neurovascular pathologically studied cases was small, limiting precision. Additional vasculo-anatomic studies of the mechanisms of symptomatic lacunar stroke are strongly needed and may soon be able to be undertaken using 7 Tesla and higher field MR imaging (41–44). Second, Fisher’s neuropathologic studies were performed more than 50 years ago when the profile of stroke risk factors and their control in the US population were very different from the present. Since then, hypertension management, statin therapy, antithrombotic treatment, and vascular risk factor profiles have changed considerably. Consequently, the relative contribution of microatherosclerosis, intracranial branch ostial atherosclerosis, lipohyalinosis, and embolism to cerebral small vessel infarcts today may differ among contemporary patients from historical observations. Third, Fisher’s studies were performed predominantly a population in the Northeastern United States. The distribution of vascular stroke mechanisms is likely to differ in other race-ethnic groups; indeed intracranial branch origin atheromatosis is known to be more common in Asian populations (45). Fourth, the study protocol was not registered prospectively. Fifth, while the risk of bias ratings were almost uniformly low for studies included in two of the meta-analyses, a modest proportion (7 of 23) of the studies contributing to the estimate of the proportion of ischemic strokes due to lacunar infarcts had high risk of bias. However, the estimands in these studies were equally split between being lower (3), higher (3), or aligned (1) with the overall estimate so had little net effect on findings.

## Conclusion

Microatherosclerotic plaque fully confined to a segment of a small, penetrating, cerebral artery was estimated to be a common cause of cerebral ischemia, underlying 1 of every 2.5 lacunar strokes and 1 in 12 of all ischemic strokes. This frequent etiologic presence of microatherosclerosis is likely an important pathophysiologic basis for the benefits of thrombolytic and statin therapy in select patients with lacunar ischemic strokes and may identify patients with distinctive, enhanced benefit from these interventions.

## Data Availability

The original contributions presented in the study are included in the article/Supplementary material, further inquiries can be directed to the corresponding author/s.
