# Supplementary material for: Microatherosclerosis is a common but often overlooked cause of ischemic stroke
Source: Front Neurol. 2026 Oct 5;17:1920003. doi: 10.3389/fneur.2026.1920003 (PMC13677506; doi:10.3389/fneur.2026.1920003)
Supplement: Supplementary file 1 [file Data_Sheet_1.PDF]

Supplement To

Microatherosclerosis is a Common but Often Overlooked Cause of Ischemic Stroke

Table of Contents

Supplemental Methods Text S1: Detailed Methods for the Three Meta-Analyses

Supplemental Table S1. Risk of Bias Ratings for Lacunar Stroke among All Ischemic Strokes

Supplemental Table S2. Risk of Bias Ratings for Lacunar Stroke Clinical Syndromes among All Lacunar Strokes

Supplemental Table S3. Etiologic Findings Upon Serial Sectioning of Penetrating Arteries During Post-Mortem Neuropathologic Examination of Patients with Histories of Different Clinical Lacunar Stroke Syndromes

Supplemental Table S4a: Sensitivity Analysis of Frequencies of Neuropathologic Substrates Among Lacunar Strokes, Varying the Rate of Lacunar Infarcts as the Cause of Sensorimotor Stroke Syndromes

Supplemental Table S4b: Sensitivity Analysis of Frequencies of Small Vessel Neuropathologic Substrates Among All Ischemic Strokes, Varying the Rate of Lacunar Infarcts as the Cause of Sensorimotor Stroke Syndromes

Supplemental Table S5a: Sensitivity Analysis of Frequencies of Neuropathologic Substrates Among Lacunar Strokes with Variations in the Pathologic Distribution of "Other" Lacunar Syndromes

Supplemental Table S5b: Sensitivity Analysis of Frequencies of Neuropathologic Substrates Among All Ischemic Strokes with Variations in the Pathologic Distribution of "Other" Lacunar Syndromes

Supplement Figure S1. Forest Plot demonstrating Proportion of Lacunar Stroke Among All Ischemic Strokes.

Supplemental Figure S2. Donut chart showing frequency of distinct lacunar syndromes among all clinical lacunar strokes

Supplemental Methods Text S1. Detailed Methods for the Three Meta-Analyses

Meta-Analysis of the Frequency of Lacunar Stroke Among All Ischemic Strokes

We conducted a systematic review and meta-analysis of the frequency of lacunar ischemic stroke subtype among all ischemic strokes. The study adhered to the Preferred Reporting Items for Systematic Reviews and Meta-Analyses (PRISMA) guidelines[1].

Data Sources & Searches

Studies published before 2024 were identified by extraction from a recent meta-analysis of investigations of ischemic stroke: ["lacunar stroke" OR “small vessel disease” OR “stroke subtype”] AND [“TOAST” OR “ASCOD” OR "Causative Classification System”]. Reference lists of retrieved articles and previous systematic reviews were screened for additional relevant studies.

Study Selection

Criteria for inclusion were: (1) formal classification of ischemic stroke subtypes in consecutive patients performed using any of 3 validated classification systems: TOAST (Trial of ORG 10172 in Acute Stroke Treatment); ASCOD (atherosclerosis, small vessel disease, cardiac pathology, other causes, and dissection); or Causative Classification System[3],[4],[5] and (2) studies reported the frequencies of each of five causes: large artery atherosclerosis, SVD, cardioembolism, other defined cause, and undetermined/cryptogenic. One reviewer (SW) screened all listed studies, and a second reviewer (JLS) screened studies deemed probably or possibly salient by the first reviewer, with the final decision by consensus if disagreement occurred.

Data Extraction

Two investigators (SW and JLS) independently extracted data from the included studies, including study name, publication year, sample size, and the frequency of six lacunar stroke clinical syndrome subtypes: 1) pure motor hemiparesis (PMH); 2) pure sensory stroke (PSS); 3) dysarthria-clumsy hand syndrome (DCH); 4) ataxic-hemiparesis (AH); 5) sensorimotor stroke (SMS); and 6) Other. Discrepancies between reviewers were resolved by a re-review and consensus discussion.

Quality Assessment Using ROBINS-E

The quality of included observational studies was assessed using the ROBINS-E tool[6]. This assessment evaluated seven key domains for potential bias due to: confounding, participant selection, arising from measurement of exposure, post-exposure interventions, missing data, measurement of outcome, selection of reported results.

Each study was rated as having low, moderate, serious, or critical risk of bias by two independent raters (SW and JLS) with discrepancies resolved through discussion. The overall risk of bias was determined by the highest level of bias identified across all domains.

Meta-Analysis of the Relative Frequency of Classical Lacunar Stroke Clinical Syndromes among All Lacunar Strokes

An additional meta-analysis was performed according to PRISMA recommendations to determine the relative frequencies of the four classic lacunar syndromes, pure motor hemiparesis (PMH), pure sensory stroke (PSS), dysarthria-clumsy hand (DCH), and ataxic hemiparesis (AH), as well as other (OTH), among all lacunar ischemic strokes.

Data Sources & Search Strategy

We systematically searched PubMed to identify relevant clinical studies published between January 1985 to July 2024. The search strategy was: ["lacunar stroke" OR "lacunar syndrome" OR "lacunar infarct" OR "pure motor hemiparesis" OR "pure sensory stroke" OR "dysarthria-clumsy hand" OR "ataxic hemiparesis" OR "sensorimotor"] and ["epidemiology" OR "clinical series" OR "consecutive"]

Study Selection

Eligible studies met the following inclusion criteria: 1) moderate to large (≥ 10 patients) clinical series of consecutive patients; 2) reporting of the proportion of lacunar strokes due to pure motor hemiparesis, pure sensory stroke, dysarthria-clumsy hand, and/or ataxic hemiparesis. Exclusion criteria included studies focusing on non-lacunar stroke subtypes, studies not providing information on the syndromic subtypes of lacunar stroke, or those without original data (e.g., review articles). Study selection was performed in two stages, with abstracts screened initially and full texts reviewed for final inclusion by two independent reviewers (SW and JLS). Discrepancies were resolved through discussion.

Data Extraction

Two investigators (SW and JLS) independently extracted data from the included studies, including study name, publication year, sample size, and the frequency of lacunar stroke subtypes (PMH, PSS, DCH, AH, and OTH). Discrepancies between reviewers were resolved through discussion.

Quality Assessment Using ROBINS-E

The quality of included observational studies was assessed using the ROBINS-E tool. This assessment evaluated seven key domains for potential bias due to: confounding, participant selection, arising from measurement of exposure, post-exposure interventions, missing data, measurement of outcome, selection of reported results.

Each study was rated as having low, moderate, serious, or critical risk of bias by two independent raters (SW and JLS) with discrepancies resolved through discussion. The overall risk of bias was determined by the highest level of bias identified across all domains.

<u>Meta-Analysis of Pathologically Identified Causes of Lacunar Stroke</u>

An additional meta-analysis was performed according to PRISMA recommendations to quantify the pathologically-identified mechanisms of lacunar stroke associated with each lacunar clinical syndrome, with particular attention to the role of microatherosclerosis. Any mechanism mentioned once or more in the studies was included as a pathophysiologic category, which resulted in five distinct categories (Figure 2): [7],[8]

1) *Lipohyalinosis*: refers to a small-vessel vasculopathy involving penetrating arteries typically 40–200 μm in diameter[7]. It is characterized by fibrinoid necrosis, hyaline and lipid deposition, and mural thickening. Different sources identify lipohyalinosis as

identical to, partially overlapping with, or different from arteriolosclerosis. For this study, we considered arteriosclerosi s a subset of lipohyalinosis, present when the affected vessel was in the size range of arterioles (10-100 μm).

2) *Microatherosclerosis*: refers to atheromatous plaque formation within penetrating arteries typically 200–800 μm in diameter[7]. The stages of atherosclerotic plaque development include: adaptive intimal thickening; fatty streak; atheroma; fibroatheroma or atheroma with thick fibrous cap; and complicated plaques with surface defects and/or hematoma, hemorrhage, and/or thrombosis[9]. A defining feature of microatherosclerosis is that the plaque is confined entirely within the penetrating artery and does not extend into the parent artery.

3) *Intracranial branch ostial atherosclerosis*: refers to the development of atherosclerotic plaque at the junction where a small penetrating artery arises from a larger parent intracranial vessel[10]. This process leads to narrowing or occlusion of the branch ostium, impairing distal perfusion.

4) *Embolism*: refers to occlusion of a penetrating artery by material (usually a thrombus) originating from a distant site, such as a proximal artery or the heart and typically involves larger penetrating arteries (200–850 μm)[7].

Data Sources & Search Strategy

We systematically searched MEDLINE from January 1966 to July 2024 and OLDMEDLINE from January 1946 to December 1965 to identify relevant neuropathologic studies. The search strategy was: ["lacunar stroke" OR pure motor hemiparesis" OR "pure sensory stroke" OR "dysarthria-clumsy hand" OR "ataxic hemiparesis" OR “sensorimotor”] AND [“neuropathology” OR "microatherosclerosis" OR "lipohyalinosis" OR “atherosclerosis” OR “arteriolosclerosis"]. Reference lists of retrieved articles were screened for additional relevant studies.

Study Selection

Eligible studies met the following inclusion criteria: 1) postmortem neuropathologic studies providing detailed analysis of a rterial vessel pathology in a series (two or more) of patients with lacunar stroke associated with one or more of the five cardinal lacunar syndromes (pure motor hemiparesis, pure sensory stroke, dysarthria-clumsy hand, ataxic hemiparesis, and sensorimotor stroke). Exclusion criteria included studies lacking vessel analysis, focusing on non-lacunar stroke subtypes, single case reports, or those without original data (e.g., review articles). Study selection was performed in two stages, with abstracts screened initially and full texts reviewed for final inclusion by two independent reviewers. Discrepancies were resolved through discussion.

Data Extraction

Two investigators (SW and JLS) independently extracted data from the included studies, including study name, publication year, sample size, vessel assessment modality (pathology or high spatial resolution in-vivo imaging), etiologies of lacunar infarcts (microatherosclerosis, lipohyalinosis, intracranial branch ostial atherosclerosis, embolism, other). Discrepancies between revi ewers were resolved through consensus.

While neuropathologic studies of the lesions underlying PMH, PSS, DCH, and AH were identified, no study was found for SMS. Imaging studies have indicated that sensorimotor stroke can arise not only from single, penetrating artery infarcts but also infarcts of multiple, adjoining penetrating arteries with ostial occlusion due to parent vessel thromboembolic occlusion[11],[12]. Accordingly, for the epidemiologic projections, half of SMS cases were considered to be lacunar (single penetrator) in nature. As the infarcts in single penetrator SMS are larger in size, similar to those of PMH, the SMS single penetrator infarcts were projected to have similar rates of causative lesions as PMH. Also, no study was found for OTH. To minimize assumptions, patients with OTH clinical syndromes were projected have the same proportion of causative vascular lesions as for the 4 classic lacunar syndromes.

Quality Assessment Using ROBINS-E

The quality of included observational studies was assessed using the ROBINS-E tool. This assessment evaluated seven key domains for potential bias due to: confounding, participant selection, arising from measurement of exposure, post-exposure interventions, missing data, measurement of outcome, selection of reported results.

Each study was rated as having low, moderate, serious, or critical risk of bias by two independent raters (SW and JLS) with discrepancies resolved through discussion. The overall risk of bias was determined by the highest level of bias identified across all domains.

Supplemental Table S1. Risk of Bias Ratings for Lacunar Stroke among All Ischemic Strokes

Risk of Bias Domains

Study	Confounding	Participant Selection	Measurement of Exposure	Post-exposure Intervention	Missing Data	Measurement of Outcome*	Selection of Reported Results	Overall
Akhtar et al 2024[13]	Low risk	Low risk	Lowe risk	Low risk	High risk	n/a	Low risk	High risk
Kijpaisalratana et al 2023[14]	Some concerns	Low risk	Low risk	Low risk	Some concerns	n/a	Low risk	High risk
Sposato et al 2012[15]	Low risk	Low risk	Low risk	Low risk	Low Risk	n/a	Low risk	Low risk
Moulin et al 2000[16]	Low risk	Low risk	Low risk	Low risk	Some concerns	n/a	Low risk	Some concerns
Perera et al 2016[17]	Some concerns	Low risk	Low risk	Low risk	Some concerns	n/a	Low risk	High risk
Ladeira et al 2015[18]	Some Concerns	Low Risk	Low Risk	Low Risk	Low Risk	n/a	Low Risk	Some Concerns
Takasugi et al 2015[19]	Low risk	Low risk	Low risk	Low risk	High risk	n/a	Low risk	High risk
Putaala et al 2015[20]	Low risk	Low risk	Low risk	Low risk	Low risk	n/a	Low risk	Low risk
Bang et al 2003[21]	Low risk	Low risk	Low risk	Low risk	Some concerns	n/a	Low risk	Some concerns
Soda et al 2004[22]	Low risk	Low risk	Low risk	Low risk	Low risk	n/a	Low risk	Low risk
Michel et al 2010[23]	Some concerns	Low Risk	Low Risk	Low Risk	Some concerns	n/a	Low Risk	High risk

Li et al 2015[24]	Low risk	Low risk	Low risk	Low risk	Some concerns	n/a	Low risk	Some concerns
Ntaios et al 2015[25]	Low risk	Low risk	Low risk	Low risk	High risk	n/a	Low risk	High risk
Acciarresi et al 2010[26]	Low risk	Low risk	Low risk	Low risk	Low risk	n/a	Low risk	Low risk
Grau et al 2001[27]	Low risk	Low risk	Low risk	Low risk	Low risk	n/a	Low risk	Low risk
Kolominsky Rabas et al 2001[28]	Low risk	Low risk	Low risk	Low risk	Some concerns	n/a	Low risk	Some concerns
Shang et al 2011[29]	Low risk	Low risk	Low risk	Low risk	Some concerns	n/a	Low risk	Some concerns
Vallejos et al 2012[30]	Low risk	Low risk	Low risk	Low risk	Some concerns	n/a	Low risk	Some concerns
Romano et al 2013[31]	Low risk	Low risk	Low risk	Low risk	Some concerns	n/a	Low risk	Some concerns
Wolf et al 2011[32]	Low risk	Low Risk	Low Risk	Low Risk	Some concerns	n/a	Low Risk	Some concerns
Murat Sumer et al 2002[33]	Some concerns	Low Risk	Low Risk	Low Risk	Some concerns	n/a	Low Risk	High Risk
Marnane et al 2010[34]	Low risk	Low Risk	Low Risk	Low Risk	Low risk	n/a	Low Risk	Low risk
Nam et al 2012[35]	Low risk	Low risk	Low risk	Low risk	Some concerns	n/a	Low risk	Some concerns
Etgen et al 2013[36]	Some concerns	Low risk	Low risk	Low risk	Some concerns	n/a	Low risk	High risk

*Domain 6: Not applicable to baseline studies

Supplemental Table S2: Risk of Bias Ratings for Lacunar Stroke Clinical Syndromes among All Lacunar Strokes

Study	Confounding	Participant Selection	Measurement of Exposure	Post-exposure Intervention	Missing Data	Measurement of Outcome*	Selection of Reported Results	Overall
Arboix et al 2010[37]	Low risk	Low risk	Low risk	Low risk	Low Risk	n/a	Low risk	Low risk
Chamorro et al 1991[38]	Some concerns	Low risk	Low risk	Low risk	Low Risk	n/a	Low risk	Some Concerns
Besson et al 2000[39]	Low Risk	Low risk	Low risk	Low risk	Low Risk	n/a	Low risk	Low Risk
Micheli et al 2008[40]	Low Risk	Low risk	Low risk	Low risk	Low Risk	n/a	Low risk	Low Risk
Hommel et al 1990[41]	Low Risk	Low risk	Low risk	Low risk	Low Risk	n/a	Low risk	Low Risk
Gan et al 1997[42]	Low Risk	Low risk	Low risk	Low risk	Low Risk	n/a	Low risk	Low Risk
Schonewille 1999[43]	Low Risk	Low risk	Low risk	Low risk	Low Risk	n/a	Low risk	Low Risk

*Domain 6: Not applicable to baseline studies

Supplemental Table S3. Etiologic Findings Upon Serial Sectioning of Penetrating Arteries During Post-Mortem Neuropathologic Examination of Patients with Histories of Different Clinical Lacunar Stroke Syndromes

Lacunar Stroke Syndrome	Neuropathologic Cases (n)	Frequencies of Neuropathologic Substrates Underlying Each Lacunar Infarct Syndrome*					
		Microathero-sclerosis	Lipohyalinosis	Intracranial Branch Ostial Atherosclerosis	Embolism	Microaneurysm thrombosis	Inconclusive
PMH	14	9	2	12	--	--	--
PSS	2	--	2	--	--	--	--
DCH/AH/Other	3	1	--	1	1	--	--
SMS	1	--	--	--	--	--	1
Syndrome Not Described	2	--	--	--	1	1	--

PMH = pure motor hemiparesis; PSS = pure sensory stroke; DCH = dysarthria clumsy hand; AH = ataxic hemiparesis; SMS = sensori motor stroke

Supplemental Table S4a: Sensitivity Analysis of Frequencies of Neuropathologic Substrates Among Lacunar Strokes, Varying the Rate of Lacunar Infarcts as the Cause of Sensorimotor Stroke Syndromes

	Percent of SMS Clinical Syndromes Due to Lacunar Infarcts*	Frequencies of Neuropathologic Substrates Underlying Each Lacunar Infarct Syndrome*			
		Microatherosclerosis	Lipohyalinosis	Intracranial Branch Ostium Atherosclerosis*	Embolism
Frequency of Neuropathologic Substrates Among All Lacunar Ischemic Strokes	30%	43.3%	20.5%	15.8%	19.2%
	50%	43.0%	19.7%	17.1%	20.2%
	60%	42.3%	18.6%	17.4%	20.4%

*Estimand was 50%. Sensitivity analyses explored a low value of 30% and high value of 60%.

Supplemental Table S4b: Sensitivity Analysis of Frequencies of Small Vessel Neuropathologic Substrates Among All Ischemic Strokes, Varying the Rate of Lacunar Infarcts as the Cause of Sensorimotor Stroke Syndromes

	Percent of SMS Clinical Syndromes Due to Lacunar Infarcts*	Frequencies of Small Vessel Neuropathologic Substrates Underlying Each Lacunar Infarct Syndrome*			
		Microatherosclerosis	Lipohyalinosis	Intracranial Branch Ostium Atherosclerosis*	Embolism
Frequency of Small Vessel Neuropathologic Substrates Among All Ischemic Strokes	30%	7.7%	3.7%	2.8%	3.4%
	50%	7.6%	3.4%	3%	3.6%
	60%	7.5%	3.3%	3.1%	3.6%

*Estimand was 50%. Sensitivity analyses explored a low value of 30% and high value of 60%.

Supplemental Table S5a: Sensitivity Analysis of Frequencies of Neuropathologic Substrates Among Lacunar Strokes with Variations in the Pathologic Distribution of "Other" Lacunar Syndromes

	Frequencies of Neuropathologic Substrates Underlying Each Lacunar Infarct Syndrome*			
	**Microatherosclerosis**	**Lipohyalinosis**	**Intracranial Branch Ostium Atherosclerosis***	**Embolism**
Lead analysis: combined 4 classical syndromes (25% each)	42.6%	19.2%	16.9%	20.0%
Causes of PMH only	46.1%	18.3%	15.3%	19.1%
Causes of PSS only	40.4%	25.9%	14.7%	17.8%
Causes of combined DCH/AH only	43.3%	17.0%	17.6%	20.7%

*Estimand was 50%. Sensitivity analyses explored a low value of 30% and high value of 60%.

Supplemental Table S5b: Sensitivity Analysis of Frequencies of Neuropathologic Substrates Among All Ischemic Strokes with Variations in the Pathologic Distribution of "Other" Lacunar Syndromes

	**Frequencies of Neuropathologic Substrates Underlying Each Lacunar Infarct Syndrome***			
	**Microatherosclerosis**	**Lipohyalinosis**	**Intracranial Branch Ostium Atherosclerosis***	**Embolism**
Lead analysis: combined 4 classical syndromes (25% each)	7.6%	3.4%	3.0%	3.6%
Causes of PMH only	8.2%	3.3%	2.7%	3.4%
Causes of PSS only	7.2%	4.6%	2.6%	3.2%
Causes of combined DCH/AH only	7.7%	3.0%	3.1%	3.7%

Values represent the percentage of all ischemic strokes, based on the meta-analytic estimate that lacunar infarcts account for 17.8% of all ischemic strokes.

*Estimand was 50%. Sensitivity analyses explored a low value of 30% and high value of 60%.

Supplement Figure S1. Forest Plot demonstrating Proportion of Lacunar Stroke Among All Ischemic Strokes.

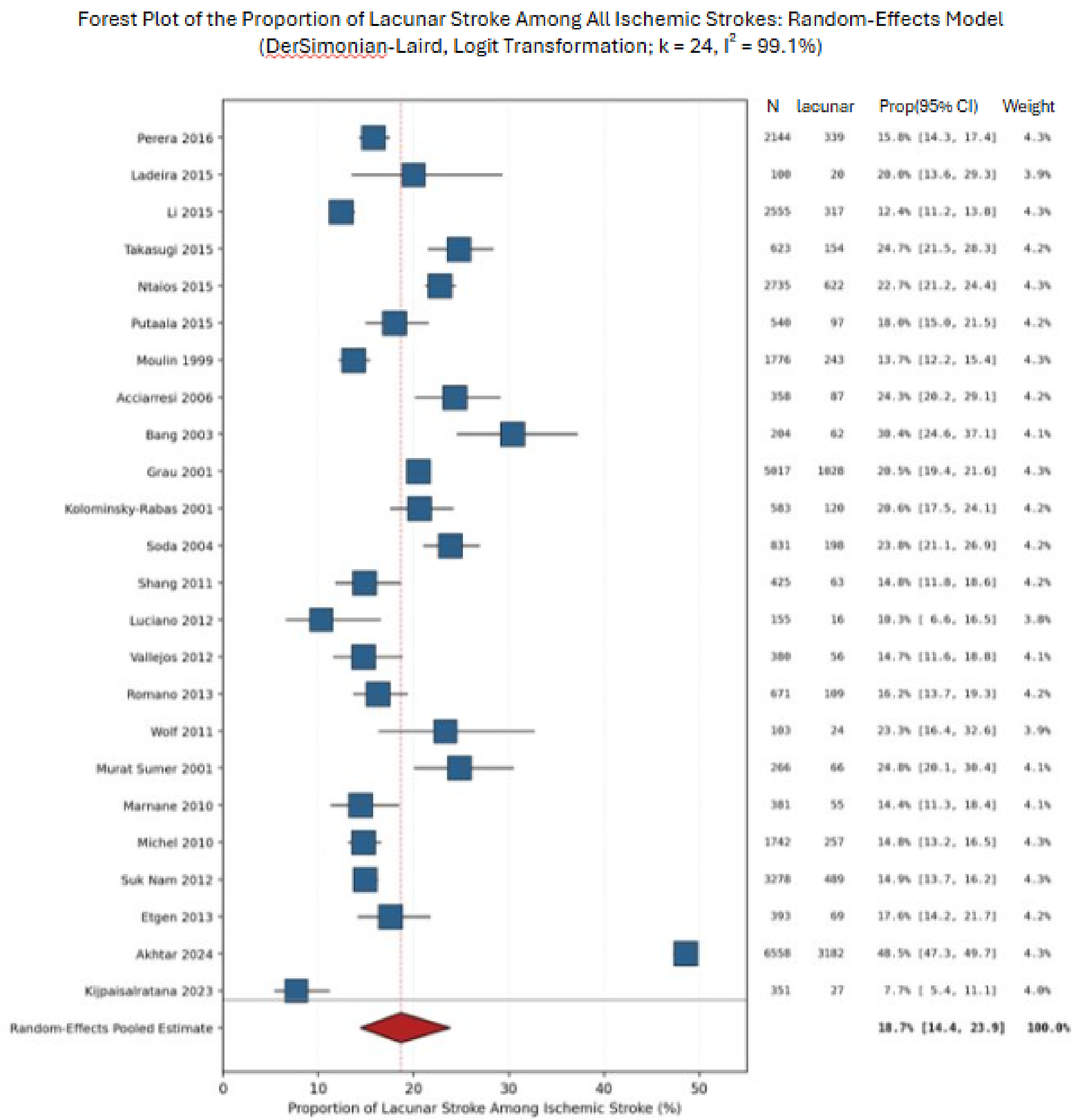

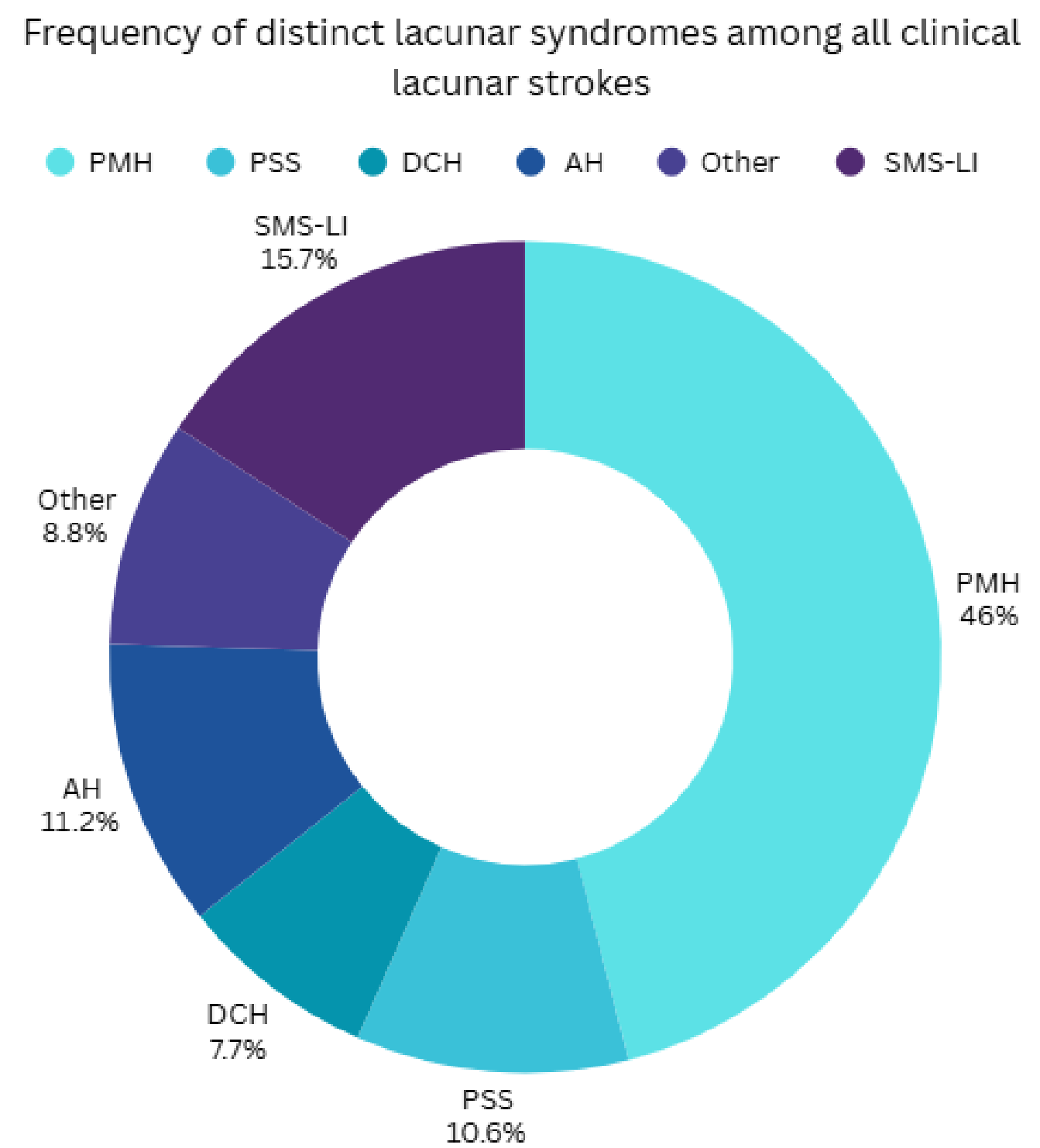

Supplemental Figure S2: Donut chart showing frequency of distinct lacunar syndromes among all clinical lacunar strokes

The study weighted frequencies of distinct lacunar syndromes among all clinical lacunar strokes were PMH (47%), PSS (10.6%), DCH (7.7%), AH (11.2%), other (8.8%), and SMS-LI (15.7%). The PMH total is adjusted because each value was rounded to a single decimal place. PMH indicates pure motor hemiparesis; PSS, pure sensory stroke; DCH, dysarthria-clumsy hand; AH, ataxic hemiparesis; SMS-LI, sensorimotor stroke- lacunar infarct.

REFERENCES for Studies Included in Meta-Analyses

1. Page MJ, McKenzie JE, Bossuyt PM, et al. The PRISMA 2020 statement: an updated guideline for reporting systematic reviews. *BMJ*. 2021;372:n71. doi:10.1136/bmj.n71

2. Sung EM, Saver JL. Statin Overuse in Cerebral Ischemia Without Indications: Systematic Review and Annual US Burden of Adverse Events. *Stroke*. 2024;55(8):2022-2033. doi:10.1161/STROKEAHA.123.044071

3. Adams HP, Bendixen BH, Kappelle LJ, et al. Classification of subtype of acute ischemic stroke. Definitions for use in a multi center clinical trial. TOAST. Trial of Org 10172 in Acute Stroke Treatment. *Stroke*. 1993;24(1):35-41. doi:10.1161/01.str.24.1.35

4. Amarenco P, Bogousslavsky J, Caplan LR, Donnan GA, Wolf ME, Hennerici MG. The ASCOD phenotyping of ischemic stroke (Updated ASCO Phenotyping). *Cerebrovasc Dis Basel Switz*. 2013;36(1):1-5. doi:10.1159/000352050

5. Ay H, Furie KL, Singhal A, Smith WS, Sorensen AG, Koroshetz WJ. An evidence-based causative classification system for acute ischemic stroke. *Ann Neurol*. 2005;58(5):688-697. doi:10.1002/ana.20617

6. Higgins JPT, Morgan RL, Rooney AA, et al. A tool to assess risk of bias in non-randomized follow-up studies of exposure effects (ROBINS-E). *Environ Int*. 2024;186:108602. doi:10.1016/j.envint.2024.108602

7. Miller Fisher C. Lacunar Infarcts – A Review. *Cerebrovasc Dis*. 1991;1(6):311-320. doi:10.1159/000108861

8. Blanco-Ruiz M, Saver JL. Distinctive feeding vessel architecture and sparse collateralization may underlie the characteristic early temporal pole and external capsule white matter compromise in CADASIL. *Med Hypotheses*. 2024;183:111267. doi:10.1016/j.mehy.2024.111267

9. Stary HC. Natural history and histological classification of atherosclerotic lesions: an update. *Arterioscler Thromb Vasc Biol*. 2000;20(5):1177-1178. doi:10.1161/01.atv.20.5.1177

10. Caplan LR. Intracranial branch atheromatous disease: a neglected, understudied, and underused concept. *Neurology*. 1989;39(9):1246-1250. doi:10.1212/wnl.39.9.1246

11. Adams HP, Damasio HC, Putman SF, Damasio AR. Middle cerebral artery occlusion as a cause of isolated subcortical infarction. *Stroke*. 1983;14(6):948-952. doi:10.1161/01.str.14.6.948

12. Arboix A, Massons J, García-Eroles L, Targa C, Comes E, Parra O. Clinical predictors of lacunar syndrome not due to lacunar infarction. *BMC Neurol*. 2010;10:31. doi:10.1186/1471-2377-10-31

13. Akhtar N, Singh R, Kamran S, et al. Association between serum triglycerides and stroke type, severity, and prognosis. Analysis in 6558 patients. *BMC Neurol*. 2024;24(1):88. doi:10.1186/s12883-024-03572-9

14. Kijpaisalratana N, Ament Z, Bevers MB, et al. Trimethylamine N-Oxide and White Matter Hyperintensity Volume Among Patients With Acute Ischemic Stroke. *JAMA Netw Open*. 2023;6(8):e2330446. doi:10.1001/jamanetworkopen.2023.30446

15. Sposato LA, Klein FR, Jáuregui A, et al. Newly diagnosed atrial fibrillation after acute ischemic stroke and transient ischemic attack: importance of immediate and prolonged continuous cardiac monitoring. *J Stroke Cerebrovasc Dis Off J Natl Stroke Assoc*. 2012;21(3):210-216. doi:10.1016/j.jstrokecerebrovasdis.2010.06.010

16. Moulin T, Tatu L, Vuillier F, Berger E, Chavot D, Rumbach L. Role of a stroke data bank in evaluating cerebral infarction subtypes: patterns and outcome of 1,776 consecutive patients from the Besançon stroke registry. *Cerebrovasc Dis Basel Switz*. 2000;10(4):261-271. doi:10.1159/000016068

17. Perera KS, Vanassche T, Bosch J, et al. Embolic strokes of undetermined source: Prevalence and patient features in the ESUS Global Registry. *Int J Stroke Off J Int Stroke Soc*. 2016;11(5):526-533. doi:10.1177/1747493016641967

18. Ladeira F, Barbosa R, Caetano A, Mendonça MD, Calado S, Viana-Baptista M. Embolic stroke of unknown source (ESUS) in young patients. *Int J Stroke Off J Int Stroke Soc*. 2015;10 Suppl A100:165. doi:10.1111/ijs.12596

19. Takasugi J, Yamagami H, Toyoda K, Nagatsuka K. Diagnostic considerations of embolic strokes of undetermined source on admission. *Neurosonology*. 2015;28:17-20. doi:10.2301/neurosonology.28.17

20. Putaala J, Nieminen T, Haapaniemi E, et al. Undetermined stroke with an embolic pattern--a common phenotype with high early recurrence risk. *Ann Med*. 2015;47(5):406-413. doi:10.3109/07853890.2015.1057612

21. Bang OY, Lee PH, Joo SY, Lee JS, Joo IS, Huh K. Frequency and mechanisms of stroke recurrence after cryptogenic stroke. *Ann Neurol*. 2003;54(2):227-234. doi:10.1002/ana.10644

22. Soda T, Nakayasu H, Maeda M, et al. Stroke recurrence within the first year following cerebral infarction--Tottori University Lacunar Infarction Prognosis Study (TULIPS). *Acta Neurol Scand*. 2004;110(6):343-349. doi:10.1111/j.1600-0404.2004.00290.x

23. Michel P, Odier C, Rutgers M, et al. The Acute STroke Registry and Analysis of Lausanne (ASTRAL): design and baseline analysis of an ischemic stroke registry including acute multimodal imaging. *Stroke*. 2010;41(11):2491-2498. doi:10.1161/STROKEAHA.110.596189

24. Li L, Yiin GS, Geraghty OC, et al. Incidence, outcome, risk factors, and long-term prognosis of cryptogenic transient ischaemic attack and ischaemic stroke: a population-based study. *Lancet Neurol*. 2015;14(9):903-913. doi:10.1016/S1474-4422(15)00132-5

25. Ntaios G, Papavasileiou V, Milionis H, et al. Embolic strokes of undetermined source in the Athens stroke registry: a descriptive analysis. *Stroke*. 2015;46(1):176-181. doi:10.1161/STROKEAHA.114.007240

26. Acciarresi M, Caso V, Venti M, et al. First-ever stroke and outcome in patients admitted to Perugia Stroke Unit: predictors for death, dependency, and recurrence of stroke within the first three months. *Clin Exp Hypertens N Y N 1993*. 2006;28(3-4):287-294. doi:10.1080/10641960600549249

27. Grau AJ, Weimar C, Buggle F, et al. Risk factors, outcome, and treatment in subtypes of ischemic stroke: the German stroke data bank. *Stroke*. 2001;32(11):2559-2566. doi:10.1161/hs1101.098524

28. Kolominsky-Rabas PL, Weber M, Gefeller O, Neundoerfer B, Heuschmann PU. Epidemiology of ischemic stroke subtypes according to TOAST criteria: incidence, recurrence, and long-term survival in ischemic stroke subtypes: a population-based study. *Stroke*. 2001;32(12):2735-2740. doi:10.1161/hs1201.100209

29. Shang W yu, Liu J yan. Stroke subtype classification: a comparative study of ASCO and modified TOAST. *J Neurol Sci*. 2012;314(1-2):66-70. doi:10.1016/j.jns.2011.10.029

30. Vallejos J, Jaramillo A, Reyes A, et al. Prognosis of cryptogenic ischemic stroke: a prospective single-center study in Chile. *J Stroke Cerebrovasc Dis Off J Natl Stroke Assoc*. 2012;21(8):621-628. doi:10.1016/j.jstrokecerebrovasdis.2011.02.005

31. Romano JG, Arauz A, Koch S, et al. Disparities in stroke type and vascular risk factors between 2 Hispanic populations in Miami and Mexico city. *J Stroke Cerebrovasc Dis Off J Natl Stroke Assoc*. 2013;22(6):828-833. doi:10.1016/j.jstrokecerebrovasdis.2012.05.016

32. Wolf ME, Sauer T, Alonso A, Hennerici MG. Comparison of the new ASCO classification with the TOAST classification in a population with acute ischemic stroke. *J Neurol*. 2012;259(7):1284-1289. doi:10.1007/s00415-011-6325-1

33. Murat Sumer M, Erturk O. Ischemic stroke subtypes: risk factors, functional outcome and recurrence. *Neurol Sci Off J Ital Neurol Soc Ital Soc Clin Neurophysiol*. 2002;22(6):449-454. doi:10.1007/s100720200004

34. Marnane M, Duggan CA, Sheehan OC, et al. Stroke subtype classification to mechanism-specific and undetermined categories by TOAST, A-S-C-O, and causative classification system: direct comparison in the North Dublin population stroke study. *Stroke*. 2010;41(8):1579-1586. doi:10.1161/STROKEAHA.109.575373

35. Nam HS, Kim HC, Kim YD, et al. Long-term mortality in patients with stroke of undetermined etiology. *Stroke*. 2012;43(11):2948-2956. doi:10.1161/STROKEAHA.112.661074

36. Etgen T, Hochreiter M, Mundel M, Freudenberger T. Insertable cardiac event recorder in detection of atrial fibrillation after cryptogenic stroke: an audit report. *Stroke*. 2013;44(7):2007-2009. doi:10.1161/STROKEAHA.113.001340

37. Arboix A, Massons J, García-Eroles L, et al. Nineteen-year trends in risk factors, clinical characteristics and prognosis in lacunar infarcts. *Neuroepidemiology*. 2010;35(3):231-236. doi:10.1159/000319460

38. Chamorro A, Sacco RL, Mohr JP, et al. Clinical-computed tomographic correlations of lacunar infarction in the Stroke Data Bank. *Stroke*. 1991;22(2):175-181. doi:10.1161/01.str.22.2.175

39. Besson G, Hommel M, Perret J. Risk factors for lacunar infarcts. *Cerebrovasc Dis Basel Switz*. 2000;10(5):387-390. doi:10.1159/000016095

40. Micheli S, Agnelli G, Palmerini F, et al. Need for extensive diagnostic work-up for patients with lacunar stroke. *J Neurol*. 2008;255(5):637-642. doi:10.1007/s00415-008-0762-5

41. Hommel M, Besson G, Le Bas JF, et al. Prospective study of lacunar infarction using magnetic resonance imaging. *Stroke*. 1990;21(4):546-554. doi:10.1161/01.str.21.4.546

42. Gan R, Sacco RL, Kargman DE, Roberts JK, Boden-Albala B, Gu Q. Testing the validity of the lacunar hypothesis: the Northern Manhattan Stroke Study experience. *Neurology*. 1997;48(5):1204-1211. doi:10.1212/wnl.48.5.1204

43. Schonewille WJ, Tuhrim S, Singer MB, Atlas SW. Diffusion-weighted MRI in acute lacunar syndromes. A clinical-radiological correlation study. *Stroke*. 1999;30(10):2066-2069. doi:10.1161/01.str.30.10.2066
